# Implementation of cancer next-generation sequencing testing in a community hospital

**DOI:** 10.1101/mcs.a003707

**Published:** 2019-06

**Authors:** Yassmine Akkari, Tamara Smith, Jennifer Westfall, Stacie Lupo

**Affiliations:** Department of Molecular Diagnostics, Legacy Laboratory Services, Legacy Health, Portland, Oregon 97232, USA

## Abstract

In the current era of personalized medicine, the field of oncology is witnessing a paradigm shift in patient care that is driving a tighter integration of genomic analysis modalities in patient care decision. This is driven by the expanding category of targeted therapies that require a broader understanding of the mutational profile of patient samples to more precisely guided personalized treatment decisions. Next-generation sequencing (NGS) has proved to be of tremendous power in detecting and characterizing a broad spectrum of activating or loss-of-function mutations across many gene targets. This power of NGS also results in significant challenges related to technical expertise, bioinformatics, computing infrastructure, laboratory practices, and integration into clinical decision-making. These challenges are particularly relevant to smaller and mid-tier hospital networks that are faced with the need to modernize their clinical practices and offer their patients access to advanced genomic technologies to improve outcomes. Adoption of such personalized medicine relies on information about a patient's cancer genome and the identification of its variants. This is best achieved using NGS. However, there are challenges to the adoption of such a complex technology and workflow, especially in smaller hospital systems. This commentary summarizes key considerations and challenges related to implementation of NGS in a community hospital setting.

## INTRODUCTION

Expanding knowledge of molecular disease mechanisms has been made clinically valuable with the advance of targeted therapy and companion diagnostics, whereby the presence of a certain DNA variant would render the tumor susceptible (sensitive) to a specific drug. Recently, next-generation sequencing (NGS) has become generally accepted as the most efficient test methodology for cancer genomes, especially with the realization that molecular alterations not only initiate, but also drive tumor growth, metastasis, and eventually resistance to therapy (Bielski et al. 2018).

NGS is a breakthrough technology that renders decoding genetic information easier, faster, and less expensive than conventional methods such as Sanger sequencing. It allows the interrogation of gene panels, transcriptome, whole-exome, and whole-genome for disease-causing mutations, rendering the application of this technology more accessible to clinical care. An increase in uptake of NGS testing in clinical laboratories over the past few years has increased market demands and decreased cost (https://www.marketresearchengine.com/reportdetails/next-generation-sequencing-ngs-market). In addition, the reimbursement rate and acknowledgment of the clinical utility of NGS tests by both private and public payers has increased significantly and is supported by the clear and documented medical benefit of personalized medicine (https://www.whitehouse.gov/the-press-office/2015/01/30/fact-sheet-president-obama-s-precision-medicine-initiative; https://www.captodayonline.com/cms-coverage-policy-ngs-cancer-testing-goes-large/).

Despite the powerful capabilities of NGS to interrogate a broad spectrum of clinically relevant genetic changes in biological samples, numerous challenges exist for its successful implementation in a community hospital because of the complexity of its workflow and interpretation of sequencing data. In this commentary, we sought to share our perspectives and experience in implementing NGS testing in a molecular pathology laboratory associated with a community hospital in which sophisticated informatic resources are limited. We present an overview of developing such a program with an emphasis on the steps undertaken for the assessment of patient care needs in terms of molecular testing, capital funding, resource leverage, and test validations.

## WHO ARE WE AS A SYSTEM?

Legacy Health is a nonprofit health system with seven hospitals including a dedicated children's hospital. With 13,000 employees, the system also includes more than 70 primary care, specialty, and urgent care clinics, as well as almost 3000 providers. It is a locally owned community-based organization serving the Portland–Vancouver metropolitan area and mid–Willamette Valley with a full range of health-care services from screening and prevention to critical care. Within this entity, Legacy Laboratory Services serves both our in-patients as well as patients from outreach programs, offering general laboratory as well as specialty testing, with CLIA, CAP, and ISO 15189 accreditation.

## NGS IMPLEMENTATION IN A COMMUNITY HOSPITAL

Our NGS program at Legacy Health was initiated based on a strong conviction that health-care delivery needs to remain local. This allows our patients to be diagnosed, treated, and potentially enrolled in clinical trials closer to their homes and families.

In addition, implementation of NGS tumor testing in a clinical genomic laboratory is advantageous not only because of the significant advances made in recent years to sequencing technologies but also to our increasing knowledge of the functional property of the human genome. The range of treatment options is much greater with NGS tumor testing, particularly when standard chemotherapy proves ineffective.

The goal of implementing NGS testing locally was to create a sustainable environment in which all components of patient care remain within our geographical region. Furthermore, it allows the cultivation of local expertise in both oncology and molecular testing interpretation to better stay abreast of new technology and better serve our community. This latter facilitates and encourages a multidisciplinary approach (both within our seven-hospital system and across other medical institutions in the state of Oregon) to the treatment of cancer patients, a fact that has been shown to improve outcomes (e.g., tumor boards). Of note, Legacy Health is part of an Oregon multi-institutional cancer collaboration in which oncologists from across the city have patient care privileges in our hospitals.

From a laboratory operation perspective, bringing NGS testing in-house allowed our team to control the quality of the process as well as the workflow through a robust quality management program (part of our ISO 15189 accreditation). Moreover, determination of appropriate turnaround time for reporting results (discussed below) and ensuring the integration of the NGS results into the patient's electronic medical records (EMRs) can be insured internally. In addition, subscription to the CAP NGS proficiency testing allows us to monitor our results and make sure they are consistent with the findings from other laboratories.

Both clinical and molecular information sharing have gained momentum in the medical community. Following multiple interdisciplinary discussions that included surgeons, oncologists, radiologists, pathologists, and a molecular geneticist, we sought a system-wide commitment to promote advances in personalized medicine and adopt NGS technology to our Molecular Diagnostics laboratory.

## COMPONENTS OF A SUCCESSFUL IMPLEMENTATION

**Figure 1.**
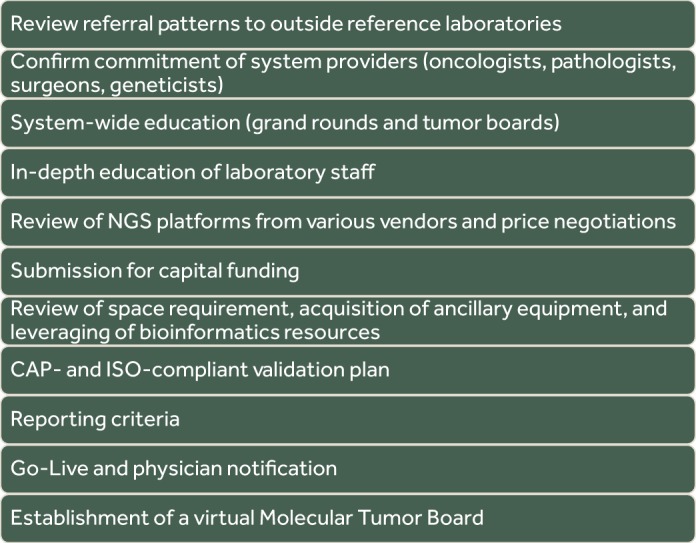
Diagram of a system planning for NGS.

The process of bringing NGS testing in-house necessitated the alignment of several processes to ensure a successful implementation (Fig. 1). Our process started with systematic review of referral patterns for NGS tests that were being sent to outside reference laboratories. Data from this review showed that NGS testing across our system was mainly performed on patients with metastatic disease when the standard therapeutic options became limited. Review of both the results and the process of sending samples for testing to commercial laboratories exposed several issues that further encouraged our approach to bringing it internally. Some of these issues related to different providers sending patient samples to different laboratories with varying limits of detection and gene coverage. In addition, there were inconsistencies in ensuring that the reports were being scanned into the patient's EMRs or that the same laboratory was being used for the same patient at different stages of disease. In addition, close review of the results from outside commercial laboratories validated our small gene panel approach as the same “culprit” genes were being detected in the most common cancers, such as colon and lung. Once the laboratory and clinical utilizations were assessed, a pattern of oncology discipline and clinical practice emerged, and the need for specific disease and gene testing became apparent. This was then aligned with our laboratory's resources and expertise. NGS applications in the clinical oncology setting range from sequencing entire tumor genomes to targeted diagnostic gene panels, which can be applied to both solid tumors and hematologic malignancies using formalin-fixed paraffin-embedded (FFPE) tissue or bone marrow/peripheral blood specimens, respectively. The number of genes included in panels can differ among testing laboratories. Panel content decisions usually relate to the establishment of clinical utility, an understanding of the ensuing economic burden on the patient and the payor, and, ultimately, the read capacity of the NGS platform validated for tumor testing. In our institution, we have adopted the GeneRead QIAct AIT DNA UMI panel, a panel of 30 actionable genes, commonly mutated in the five common cancers with high impact on population health: breast, colon, ovarian, lung, and melanoma. It is important to note that bigger hospital systems may afford to interrogate bigger gene panels based on the availability of more sophisticated bioinformatics pipelines and number of technical specialists with expertise to interpret massive amount of data. Our system, in contrast, has limited internal bioinformatics resources and needed to rely on the expertise of fewer directors for interpretation. In addition, given the smaller number of patients in our clinical setting, findings from NGS testing are likely to fall within the genes most known to be mutated in these common cancers. Patients with unusual clinical presentations and more rare cancers/gene variants will likely to be referred to an outside tertiary center for care. This necessary and sufficient gene panel testing approach has allowed us the opportunity to minimize cost and labor and offer our clinicians actionable results.

The next step was to provide in-service educational seminars to participating physicians at various tumor boards (for both germline and somatic NGS testing, respectively). These interactions gauged interest and commitment from the hospital system and referring physicians, as well as provided education opportunities to various stakeholders (including staff), to support the molecular laboratory's endeavor.

One of the biggest challenges in implementing large NGS panels is the interpretation of variants that are not found in publicly available databases and for which published functional studies are not available. Therefore, correlating these rare variants with appropriate therapies and/or establishing a direct function-to-treatment response becomes more difficult. In a small hospital system, human, informatics, and research resources for interpretation of “unknown” variants are limited, and validation of NGS panels with well-established disease genes and vetted variant hotspots has more utility and clinical impact on our patient population.

Once the above elements were established, researching the market for the NGS platform of choice was undertaken. This latter necessitated a “request for information” (RFI) from various vendors to review quality of workflow, sequencing chemistry, cost, ancillary equipment needs, and bioinformatics capabilities. These components were then matched with the resources already available in the laboratory. Next, a quote was requested and was submitted for capital funding.

Choosing from among the commercially available NGS platforms needs to be tailored to the laboratory's specific operation and can be a challenge in terms of technological sophistication and cost. Although there are several platforms to choose from, the decision should be directly related to the clinical laboratory's workflow and structure, its clinical demand, and the availability of a bioinformatics support system to enable the analysis and interpretation of the massive sequencing data generated from each run. NGS is a high-complexity test and workflow; its implementation should include review of competency of the testing personnel, in addition to ensuring additional training to provide redundancy for staff coverage.

In our institution, we decided to implement tumor NGS testing using the QIAGEN GeneReader “Sample to Insight” workflow. This technology has given us the ability to batch samples from multiple indications, helping to optimize the cost of the sequencing run. The panel was designed to detect single-nucleotide variants, small insertions and deletions, copy-number variants, and gene amplification covering well-vetted hotspots in 30 actionable genes. Our choice of the QIAGEN NGS platform was attributable to several key components: The entire system would be provided by a single vendor, allowing continuity and standardization throughout the operation, especially in relation to tracking reagent lots and software updates; our familiarity with the vendor's DNA manipulation expertise; and the availability of an integrated bioinformatics pipeline for the interpretation of the data.

Currently, we are performing NGS testing on FFPE samples from patients with metastatic disease. Our overall patient number has averaged approximately 10 to 15 patients per week performed in a single NGS run. Because our panel is pan-cancer and includes results that are potentially time-sensitive, we have set our turnaround time for 10 d from receiving the test order to the issuance of the report.

As our understanding of cancer genetics evolves and new variants are discovered, it is important to be able to access a database that contains comprehensive and up-to-date interpretation of variant significance. For example, in 2018, The College of American Pathologists (CAP), the International Association for the Study of Lung Cancer (IASLC), and the Association for Molecular Pathology (AMP) together published an evidence-based guideline that establishes recommendations for EGFR and ALK testing, helping to guide targeted therapies (Lindeman et al. 2018). We wanted to ensure the use of a database that encompasses the latest guideline information, affording patients best and proven care practice. The QIAGEN Clinical Insight Interpret (QCI-I) focuses on clinically relevant variants, approved therapeutic labels, professional association practice guidelines, and active phase 3 clinical trials. It also provided us access to a continuously updated library of information for appropriate variant interpretation.

## PLANNING AHEAD FOR IMPLEMENTATION

In any clinical laboratory, the establishment of NGS testing should be preceded by a planning algorithm whereby the laboratory determines what specimen type(s) will be tested and what diagnostic/prognostic information will be evaluated and reported. The laboratory can either choose a small set of core genes for which extensive information is available regarding targeted therapy and clinical trials or opt for a larger number of genes or target sequences in which evidence regarding pathogenicity is still growing (Jennings et al. 2017). Both approaches have benefits but following extensive discussions with our system pathologists and oncologists, our decision to use a small panel of actionable genes deemed necessary and sufficient has allowed for more direct action in clinical management and therefore a wider acceptance of the utility of our NGS test. Validation of any NGS workflow can be extensive and it is imperative that the laboratory works directly with oncologists and surgeons, participates in tumor boards to assess clinical need, helps in results interpretation, and establishes appropriate referral patterns for every patient. This was achieved in our institution through the development of a pan-cancer molecular tumor board (MOCA: Molecular Oncology and Clinical Applications).

NGS workflow is generally divided into several steps: sample preparation, target enrichment, library preparation, clonal amplification, sequencing, and sequence analysis. Implementation of clinical NGS testing requires the completion of several CAP-compliant validation processes including performance studies for accuracy, precision/reproducibility, limit of detection of variant frequencies, and analytical sensitivity/specificity. These steps should include analysis of commercially available known reference specimens and interlaboratory comparison of sequencing results and documentation of quality control metrics during each run. If a laboratory chooses to assemble individual components for a custom workflow, it is important to ensure that all steps are tested and harmonized to deliver optimal results.

## ELEMENTS OF COMPLIANCE FOR ANY NGS WORKFLOW

Compliance with CLIA and/or the CAP regulations and checklist is imperative for maintaining accreditation. Therefore, validation of NGS testing will require several steps.

1. Accuracy, precision, and reproducibility of the NGS analytics including all the wet bench components of its workflow. Adherence to these steps depends upon comparing results from patient specimens tested for the same gene using alternative molecular platforms (PCR vs. NGS), or for a panel of genes using an outside reference laboratory to establish sensitivity and specificity. In addition, inter- and intrarun comparison, as well as testing by more than one technologist, confirms reproducibility.
2. Incorporating QC metrics throughout the workflow avoids the high sequencing cost when earlier steps are compromised. Recording these exceptions can be achieved by a middleware that documents these data and tracks all tested specimens and all reagent catalog and lot numbers used throughout.
3. Establishing a quality management program including clinical reasons for referral and turnaround times and tracking patient results including correlation with reasons for referral (metastatic vs. primary cancers, resistance to therapy, etc.).
4. Validating the bioinformatics pipeline and continuous documentation of software updates. This can be achieved by processing HapMap samples in addition to known patient samples and recording software updates from the manufacturers, respectively. In addition, maintenance of privacy of the NGS data needs to be achieved and documented especially related to data storage and patient identifiers (e.g., cloud-based).

All laboratory programs seeking to implement NGS clinical testing should give strong consideration to the following five factors.

1. Integrate the experience and expertise of molecular geneticists and pathologist into the interdisciplinary tumor board.
2. Establish a testing methodology that is compatible with validated specimen types.
3. Incorporate QC metrics into NGS workflow to help mitigate the cost of sequencing.
4. Develop a robust bioinformatics pipeline for variant interpretation.
5. Ensure compliance with laboratory accreditation agencies (e.g., CAP, CLIA).

Incorporating the above steps should allow for a more efficient NGS workflow setup and a shift of emphasis in medicine from reaction to prevention. The promise of this technology includes improved diagnoses, prevention of disease progression, customized disease-prevention strategies, greater drug therapy efficacy, and avoidance of prescription drugs with predictable side effects. Last, an NGS approach will help eliminate trial-and-error inefficiencies. With the maturation of the NGS technology and selection of a workflow that best suits their need, laboratories like ours can offer locally accessible cancer care in a fast, cost-effective, and high-quality manner.

## ASSESSING SYSTEM SUCCESS

Because of the high degree of resource investment in NGS testing, it is paramount to measure the success of the implemented system with time, ideally 2 yr after the test was placed into clinical service. This can be achieved by monitoring any increase in the number of specimens being tested and/or an increase in referral patterns from various oncology clinics. In addition, internal monitoring of the number of clinical diagnoses/treatment options made possible by NGS and the identification of drug–gene interactions is recommended.

As is the case of every laboratory test, an increase in NGS workload may warrant a review of the scalability of the current NGS system in addition to updated instrument versions. It is recommended that the laboratory engages the platform vendor in these discussions in order to minimize the need to validate a different system.

## THE FUTURE OF NGS TESTING

We anticipate that NGS testing will eventually lead testing labs toward accruing genetic test results across the aggregate testing population. This will require testing laboratories to establish database capabilities to enable knowledge mining of these data sets to (1) augment the interpretation of individual test results and (2) leverage the identification of new variants to evolve the designs of the NGS tests. Furthermore, we foresee that clinical testing labs will leverage their accrued data sets to begin integration of patient clinical profiles and outcomes. This will increase the relevance of data interoperability with EMR and related patient information systems and allow hospital systems to expand their scope of interactions with pharmaceutical companies in drug development initiatives. We also anticipate that NGS data, together with orthogonal patient data, will be increasingly leveraged by Molecular Tumor Boards and clinical stakeholders involved across the patient care continuum. Of note, we recognize that liquid biopsy data sets will also be integrated into these data sets to provide longitudinal views of changes in genetic profiles of individual patients during treatment and disease progression.

As NGS testing becomes increasingly adopted in health-care provider networks, we anticipate an emergent requirement to leverage these integrated data sets to foster multi-institutional collaborations for translational research. This will naturally require industry guidelines and policies on how data rights will be secured but be able to drive harmonization of data structures to facilitate federation of data sets across institutions.

## CONCLUDING REMARKS

It has become evident that the rate of technological advances has exceeded our ability to rapidly implement the information gathered into clinical practice. We are unable to recognize the clinical significance and utility of the collected data. Increasingly, the problem we now face is how we can integrate this data into clinical practice. With an approaching wide adoption of NGS long read capability, many aspects of our genome will be uncovered, and our clinical community will have to rely on translational research before its wide implementation in routine patient care.

### Competing Interest Statement

Y.A. is a member of the QIAGEN scientific advisory board.
